# Supplementary material for: Simulating electron transfer on noisy quantum computers
Source: Nat Commun. 2026 May 28;17:4779. doi: 10.1038/s41467-026-73700-1 (PMC13219408; doi:10.1038/s41467-026-73700-1)
Supplement: Supplementary file 1 — Supplementary Information [file 41467_2026_73700_MOESM1_ESM.pdf]

Supplementary Information: Simulating Electron Transfer on Noisy Quantum Computers

Marvin Gajewski,[1,2] Alejandro D. Somoza,[1,2,*] Gary Schmiedinghoff,[3]
Pascal Stadler,[4] Michael Marthaler,[4] and Birger Horstmann[1,2,5,†]

[1] *Institute of Engineering Thermodynamics, German Aerospace Center (DLR), Wilhelm-Runge-Str. 10, 89081 Ulm, Germany*
[2] *Helmholtz Institute Ulm, Helmholtzstr. 11, 89081 Ulm, Germany*
[3] *Institute of Software Technology, German Aerospace Center (DLR), Linder Höhe, 51147 Köln, Germany*
[4] *HQS Quantum Simulations GmbH, Rintheimer Straße 23, 76131 Karlsruhe, Germany*
[5] *Department of Physics, Ulm University, Albert-Einstein-Allee 11, 89081 Ulm, Germany*

SUPPLEMENTARY NOTES

Electronic and Vibronic Electron Transfer

In Supplementary Figure 1 we show in detail the mechanisms of electronic and vibronic ET for the simulations for $N = 5$ sites that were presented in Fig. 3 in the main text.

Electronic resonances occur when the donor has similar energy to an eigenstate of the acceptor subsystem $\hat{H}_{\text{el}}^{(\text{Acc.})}$, leading to the superposition of electronic states with a mixed donor-acceptor character, as highlighted in a box in the left panel of Supplementary Figure 1. The resulting dynamics shows a slow modulation that is caused by the interference between these two eigenstates, as demonstrated in Fig. 3 a. The result in Fig. 3 a also shows fast modulations, which can be explained by the interference of these states with the ground state near $-V$ localised at the trap, which shows a small contribution of the initial donor site ($n = 0$) in Supplementary Figure 1.

Vibronic resonances occur when the donor energy Ω_0 is far-off from any acceptor states. Vibronic coupling is required in order to produce superpositions of the localized donor with delocalised acceptor states, which takes place if the energy difference is matched by the energy ω_0 of a vibrational excitation. For this reason, the charge transfer at vibronic resonances is driven by the entanglement between electronic states and quantized vibrations, forming non-separable superpositions of zero and one-phonon configurations.

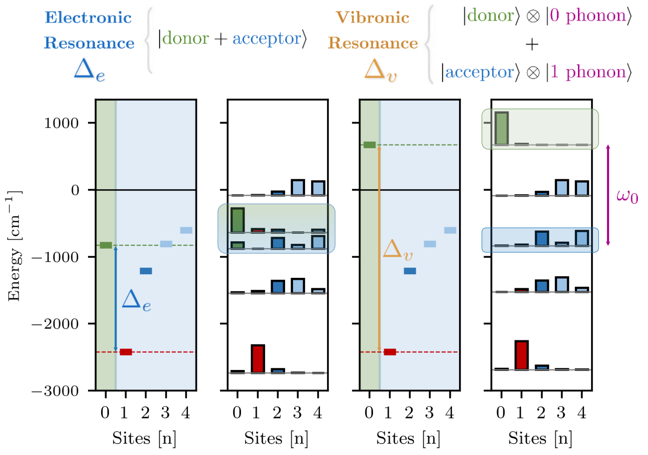

Supplementary Figure 1. **Mechanisms of purely electronic and vibronic ET for $N = 5$.** The left subpanels show the energies of the electronic Hamiltonian term $\hat{H}_{\text{el}}$ in site basis, while the right subpanels show its eigenenergies, with the amplitude of each site plotted on top of the bar. At $\Delta_e = 1435\,\text{cm}^{-1}$ (left-hand side), the donor Ω_0 is resonant with the second eigenstate of the acceptor-only electronic subsystem in Eq. 8 in the main text, producing a superposition of states with mixed donor/acceptor character. At $\Delta_v = 3010\,\text{cm}^{-1} \approx \Delta_e + \omega_0$ (right-hand side), vibronic coupling is required in order to produce superpositions of the localized donor with delocalised acceptor states.

* Corresponding author: alejandro.somoza@dlr.de
† Corresponding author: birger.horstmann@dlr.de

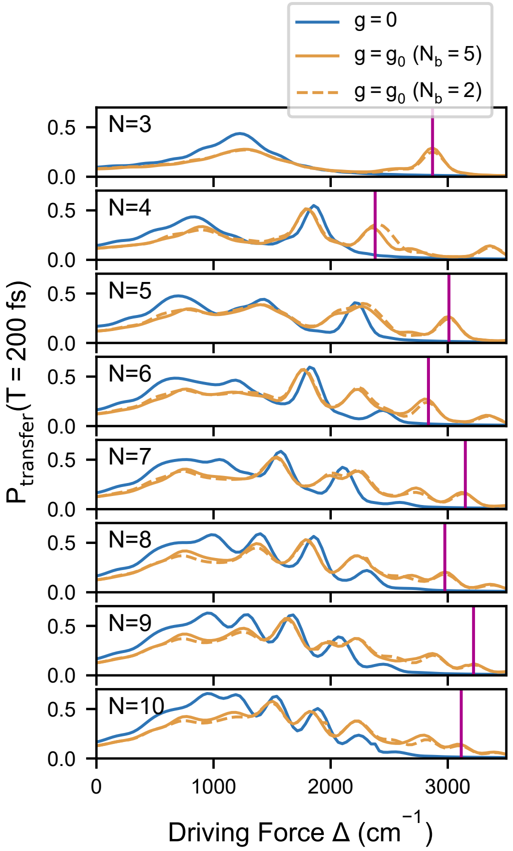

Supplementary Figure 2. **Time-averaged probability of electron transfer $P_{\rm transfer}(T = 200$ fs) as a function of the driving force for $N = 3 - 10$ sites.** We show classical simulations for the case without vibronic coupling ($g = 0$, blue) and with vibronic coupling ($g = g_0$, orange, bold). We also show a classical simulation with less levels per oscillator $N_b = 2$ (orange, dashed) to assess the truncation error. The vertical purple bars indicate the driving forces $\Delta_v(N) =$ of the respective vibronic peaks that were chosen for the simulations in this work, located at $\Delta_v(N) = 2870, 3360, 3010, 2835, 3150, 2975, 3220, 3115\,\mathrm{cm}^{-1}$ for $N = 3, 4, 5, 6, 7, 8, 9, 10$ respectively.

In Supplementary Figure 2 we show for each system size N the classically simulated transfer probabilities over the driving force, as well as for each N the driving force at which we simulated the vibronic resonances shown in Fig. 4 in the main text. For a system of N sites there are $N - 2$ vibronic resonances, of which we selected resonances with driving forces much higher than all electronic resonances, to ensure a purely vibronic mechanism.

Numerical Benchmarks and Validity of Approximations

Mapping the target Hamiltonian onto quantum circuits involves two approximations: The Trotter approximation with respect to the time step Δt, and the truncation of the oscillators to N_b energy levels. To assess the error that is introduced by each approximation, we use a third type of numerical benchmark ($\Delta t = 0.5\,\mathrm{fs}$, $N_b = 2$) together with emulations ($\Delta t = 4\,\mathrm{fs}$, $N_b = 2$ levels per oscillator) and classical simulations ($\Delta t = 0.5\,\mathrm{fs}$, $N_b = 5$). All benchmarks consider the undamped model ($\gamma = 0$). Classical simulations represent the exact dynamics, as we chose the time step Δt and the number of oscillators N_b such that both Trotter and oscillator truncation error become negligible. Hence, comparing the classical simulations to the emulations gives insights about the impact of both Trotter and truncation error together. The third type of numerical benchmark exhibits a short timestep and few oscillator levels, such that comparing them against emulations characterizes the Trotter error, and comparing them against the classical simulation with $N_b = 5$ the truncation error.

All three comparisons are shown in Supplementary Figure 3. We find that the main contributor to the full deviation is the Trotter error, which does not significantly increase with system size N. The truncation error of oscillators decreases with N, since for large models with $N > 4$, vibronic resonances with multiple vibrational excitations are

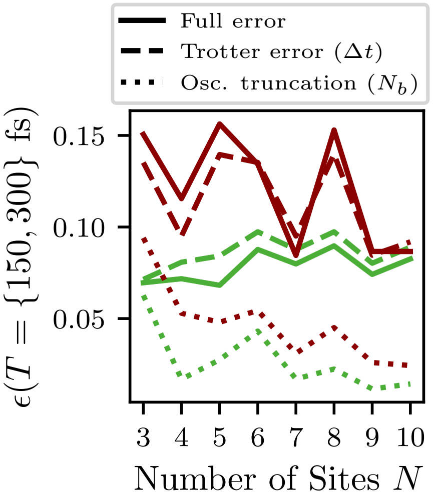

Supplementary Figure 3. **Scaling analysis for different approximations contributing to the time-averaged deviation $\epsilon(T)$.** We consider numerical benchmarks of vibronic resonances in three variations: Emulations (Type A: $\gamma = 0$, $\Delta t = 4$ fs, $N_b = 2$), short timestep with truncated oscillators ($\gamma = 0$, $\Delta t = 0.5$ fs, $N_b = 2$) and classical simulations (Type C: $\gamma = 0$, $\Delta t = 0.5$ fs, $N_b = 5$). The deviation between A and B comprises the numerical error stemming from the Trotter approximation (dashed), the deviation between B and C comprises the error due to oscillator truncation (dotted), and the deviation between A and C comprises both errors together (bold). We show the errors for $T = 150$ fs (green) and $T = 300$ fs (brown).

less probable, and the vibrational dynamics tend to stay within the subspace of a single vibrational excitation that is coherently shared among all oscillators.

In Supplementary Figure 2 we further investigate the truncation error by comparing the resonance structure of our figure of merit $P_{\text{transfer}}(\Delta)$ extracted from classical simulations with both $N_b = 2$ and $N_b = 5$ levels per oscillator, validating our quantum simulations with $N_b = 2$.

Effective Damping Rate for N=5

This section investigates the effective damping rate of the quantum simulation of the vibronic resonance shown in Fig. 3 in the main text. In Fig. 4 a we compare the experimental results on the quantum computer against classical simulations with three different damping rates. In Fig. 4 b, we plot the time-averaged deviation $\epsilon(T)$ between the experimental result and classical simulations with varying damping rates γ. The lowest deviation at $T = 200$ fs is achieved for an effective damping rate $\Gamma_{\text{QC}} = (112.5\,\text{fs})^{-1}$. While indeed the population dynamics of acceptor sites are best matched to $\Gamma_{\text{QC}} = (112.5\,\text{fs})^{-1}$, we note that the population of the donor site $P_0(t)$ exhibits a slower decay, resembling the simulation with $\gamma^{-1} = (50\,\text{fs})^{-1}$.

SUPPLEMENTARY METHODS

Qubit Mapping

We map N nearest-neighbor coupled electronic sites directly on a chain of N site qubits $\hat{\sigma}_n$ in the processor as

$$\hat{H}_{\text{el}} \rightarrow \sum_{n=0}^{N-1} \frac{\Omega_n}{2}\, \hat{\sigma}_n^z + \sum_{n=0}^{N-2} \frac{J}{2} \left(\hat{\sigma}_n^x\, \hat{\sigma}_{n+1}^x + \hat{\sigma}_n^y\, \hat{\sigma}_{n+1}^y \right). \tag{1}$$

In the following, we present the general framework for our LVC Hamiltonian model of electron transfer, where every site n couples locally to M_n oscillators. Note that the model in the main text only considers a single oscillator per site ($M_n = 1$ in $\hat{H}_{\text{vib}}$). Each local quantum harmonic oscillator in the LVC Hamiltonian is then identified by its site index n and local index m, and mapped onto an arbitrary number of Q oscillator qubits in the quantum computer.

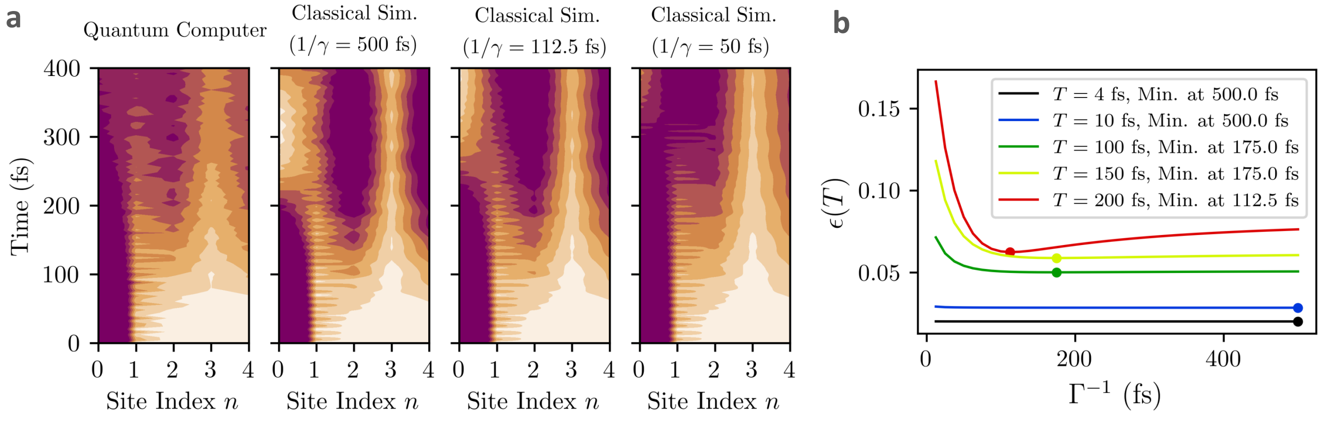

Supplementary Figure 4. **Extracting effective damping rate Γ_{QC} for $N = 5$. a.** Population dynamics at the vibronic resonance for $N = 5$ sites (5+5 qubits) executed on IBM_AACHEN on May 28, 2025, compared against classical simulations with damping rates $(500\,\text{fs})^{-1}$, $(112.5\,\text{fs})^{-1}$ and $(50\,\text{fs})^{-1}$. **b.** Time-averaged deviation $\epsilon(T)$ between experimental results and classical simulations with a fixed damping rate γ, plotted for $T = 4, 10, 100, 150$ and $200\,\text{fs}$. The minimum errors are indicated with dot markers.

We replace each of the $\hat{b}^\dagger_{n,m}\,\hat{b}_{n,m}$ bosonic operators in Eq. (3) in the main text, and coupling operators $\hat{b}^\dagger_{n,m} + \hat{b}_{n,m}$ in Eq. (4) in the main text, by a sum of Q oscillator qubit operators $\hat{\sigma}_{n,m,q}$ such that

$$\hat{b}^\dagger_{n,m}\,\hat{b}_{n,m} \to \sum_{q=0}^{Q-1} \hat{\sigma}^+_{n,m,q}\,\hat{\sigma}^-_{n,m,q},$$

$$\hat{b}^\dagger_{n,m} + \hat{b}_{n,m} \to \frac{1}{\sqrt{Q}} \sum_{m=0}^{Q-1} \hat{\sigma}^x_{n,m,q}. \tag{2}$$

This boson-to-qubit mapping introduces an error $\mathcal{O}(1/Q)$ [1] and is better suited for low excitation numbers. The vibrational part of the Hamiltonian is mapped onto qubits as

$$\hat{H}_{\text{vib}} \to \sum_{n=0}^{N-1} \sum_{m=0}^{M_n-1} \sum_{q=0}^{Q-1} \omega_{n,m}\,\hat{\sigma}^+_{n,m,q}\,\hat{\sigma}^-_{n,m,q}, \tag{3}$$

and the vibronic coupling term describing the interaction between the electronic states and oscillators is mapped onto qubits as

$$\hat{H}_{\text{el}-\text{vib}} \to \sum_{n=0}^{N-1} \sum_{m=0}^{M_n-1} \sum_{q=0}^{Q-1} g_{n,m} \left(\hat{\sigma}^z_{n,m,q} \otimes \hat{\sigma}^x_{n,m,q}\right). \tag{4}$$

Note that the number of qubits and the number of Hamiltonian terms both scale linearly in M_n and Q.

Quantum Circuits

The circuit corresponding to a single time step Δt in a first-order Trotter decomposition of the time-evolution operator $\hat{\mathcal{U}}(t) = e^{-i\hat{H}t}$ is schematically shown in Fig. 7 a in the main text. The blue box $\hat{H}_{\text{el}}$ deploys N single-qubit rotations to encode the individual energies Ω_n of system qubits and then, nearest-neighbors interactions with coupling strength J are natively corresponding to a series of $N-1$ R_{XX} and R_{YY} gates, which on IBM_AACHEN are implemented with two CZ gates and single-qubit rotations. The odd and even terms can be executed in parallel. The orange box ($\hat{H}_{\text{vib}}$) commutes with $\hat{H}_{\text{el}}$ and can be executed in parallel using N single-qubit rotations only. Finally, the magenta vibronic coupling term ($\hat{H}_{\text{el}-\text{vib}}$) can be done with a parallel cascade of N R_{XZ} gates, implemented on IBM_AACHEN with two CZ gates and single-qubit rotations, entangling each of the system qubits with their corresponding environment qubit. The number of entangling gates, depth and gate execution times depending on the system size N is given in Supplementary Table 1.

Qubits	SWAP Layers	Total CZs	Compiled with R_X Depth 1Q	Total	$T_{\text{exec.}}^{\text{Trot.}}$	Compiled with $\sqrt{X}$ Depth 1Q	Total	$T_{\text{exec.}}^{\text{Trot.}}$	Fit parameters Fig. 6 a (all fits to R_X) Min(T_1, T_2)	τ	a	b
6	0	10	12	18	0.6 µs	24	30	0.8 µs	68 µs	134 (80 µs)	0.013	0.589
8	2	20	20	32	1.2 µs	36	48	1.5 µs	95 µs	79 (95 µs)	0.084	0.382
10	2	24	20	32	1.2 µs	36	48	1.5 µs	87 µs	78 (94 µs)	0.073	0.414
12	2	28	20	32	1.2 µs	36	48	1.5 µs	87 µs	64 (77 µs)	0.083	0.405
14	2	38	20	32	1.2 µs	36	48	1.5 µs	35 µs	43 (52 µs)	0.086	0.412
16	2	42	20	32	1.2 µs	36	48	1.5 µs	63 µs	31 (37 µs)	0.113	0.384
18	2	46	20	32	1.2 µs	36	48	1.5 µs	35 µs	28 (34 µs)	0.071	0.430
20	2	54	20	32	1.2 µs	36	48	1.5 µs	35 µs	18 (22 µs)	0.101	0.338

Supplementary Table 1. **Circuit properties of a single Trotter step.** For each number of sites N and corresponding number of qubits $2N$ we display the circuit depth, number of layers of SWAP-Gates, the total number of CZ-Gates and the estimated execution time on IBM_AACHEN, based on execution times of 32 ns for single-qubit-gates and 68 ns for the CZ-gate. We did simulations using the variable-angle RX-Gate and using the SqrtX-Gate. The latter case shows longer execution times, because usually two SqrtX express one RX-Gate

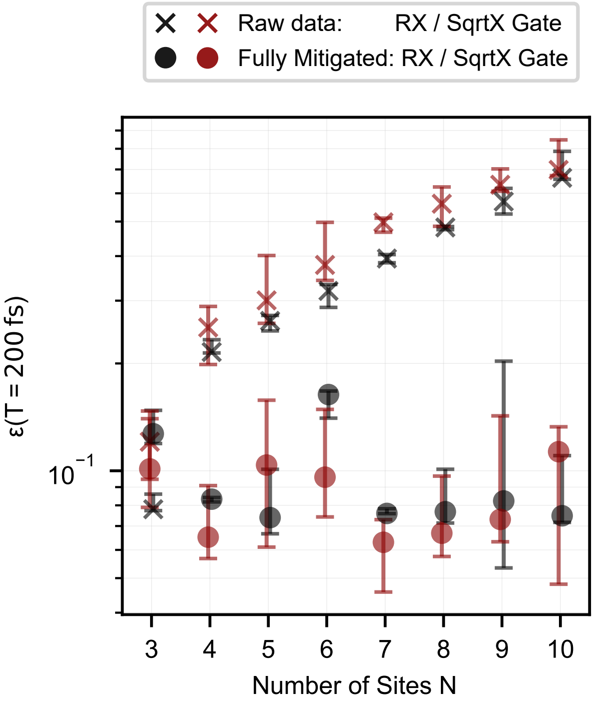

Supplementary Figure 5. **Comparison of experimental results with fixed-angle SqrtX vs. variable-angle R_X single qubit gates.** Circuits were compiled with two different types of native gates (both groups of simulations together make up the data presented in Fig. 5 a in the main text). The median, largest and smallest deviation $\epsilon(T = 200$ fs) is shown for the unprocessed and the fully mitigated data, compared against undamped emulations ($\gamma = 0$, $\Delta t = 4$ fs, $N_b = 2$).

For the experiment on real hardware, we had to adapt the quantum circuits to the heavy-hex qubit layout of the IBM Heron processor as illustrated in Fig. 1 b in the main text: the chain of sites and their attached oscillators is split into groups of three sites with their respective oscillator each. We use two SWAP gates per Trotter step to connect each of the $\lfloor N/3 \rfloor$ groups of separated sites. Note that the pairs of SWAP gates can be executed in parallel layers, such that the circuit depth does not increase with the problem size N. This enables scaling up in principle to arbitrary system sizes.

Property	$N=3$ (6 qubits) Min	Median	Max	$N=10$ (20 qubits) Min	Median	Max
1Q-Gate Error	1.1e-4	1.8e-4	2.9e-4	1.0e-4	2.0e-4	1.09e-3
2Q Gate Error	1.5e-3	1.8e-3	2.7e-3	1.2e-3	1.9e-3	5.9e-3
T_1 Sites	136 µs	218 µs	315 µs	108 µs	218 µs	333 µs
T_1 Osc.	175 µs	211 µs	274 µs	73 µs	175 µs	344 µs
T_2 Sites	167 µs	255 µs	331 µs	77 µs	211 µs	349 µs
T_2 Osc.	184 µs	218 µs	330 µs	46 µs	189 µs	349 µs
Readout Error	2.9e-3	3.6e-3	4.8e-3	2.3e-3	4.7e-3	1.84e-2

Supplementary Table 2. Calibration data of those qubits in IBM_AACHEN that were used for the simulations of population dynamics of the smallest and largest model with $N = 3, 10$ sites, averaged over all 10 simulations on different days.

Quantum Noise

The hardware noise processes we aim to exploit for the simulation of our target model are continuous noise processes and are described by a Lindblad superoperator

$$\mathcal{L}(\hat{\rho}) = \sum_{n,\alpha} \gamma_{n,\alpha} \left(\hat{L}_{n,\alpha}\, \hat{\rho}\, \hat{L}^{\dagger}_{n,\alpha} - \frac{1}{2} \{ \hat{L}^{\dagger}_{n,\alpha}\, \hat{L}_{n,\alpha}, \hat{\rho} \} \right). \tag{5}$$

The operators $\hat{L}_{n,\alpha}$ refer to noise processes acting on the n-th qubit, and the α index distinguishes between different noise processes. We give an overview which processes can be exploited for our target model in Tab. I in the main text.

In order to determine the effective noise occurring over one Trotter time step, we have to analyze the impact of noise after every gate in the Trotter circuit $\mathcal{U}_{\text{trot}}$. We model the effect of both continuous noise and gate noise together as a non-unitary operation N_j occurring locally after the execution of each idle operatation or gate G_j on the involved qubits. The effective hardware noise can be obtained by moving all noise operations to the right side of the Trotter circuit to occur after all gates, using commutation relations and the small angle approximation, yielding perturbed noise operations N'_j:

$$\begin{aligned} \mathcal{U}_{\text{trot}} &= G_1 G_2 \cdots G_k \longrightarrow G_1 N_1 G_2 N_2 \cdots G_k N_k \\ &\approx G_1 G_2 \cdots G_k N'_1 N'_2 \cdots N_k. \end{aligned} \tag{6}$$

Detailed treatments can be found in references [1, 2]. The perturbation of noise operations from N_j to N'_j are stronger, the larger the rotation angle of a gate is, and the larger the amplitude of the noise operation. This means that for example amplitude damping noise may become perturbed when using large angle gates, which is an important consideration in circuit design. In such conditions it is therefore advisable to use small-angle rotation gates like Rx or Rz preferably over large-angle gates like SqrtX or CNOT.

To test the relevance of these so-called large-angle gate errors to our simulations, we compiled the same circuits to either the large-angle SqrtX gate or the small (variable) angle Rx gate. In Supplementary Figure 5 we show a direct comparison against undamped emulations ($\gamma = 0$), revealing a trend in the mitigated data for lower deviations when using the large-angle SqrtX gate. This indicates that large-angle gate errors are less impactful for the given hardware noise, while better results for the simulations using SqrtX can be explained by the higher rate of (coherent) overrotation errors of the Rx gate, which is not corrected in our error mitigation scheme. When considering the unmitigated data, we observe a better performance with the Rx gate, which can be explained by the circuits with Rx gates being less deep, such that less noise accumulates during circuit execution.

Criteria for Qubit Selection

For selecting the hardware qubits on which we place the quantum circuit, we use Mapomatic [3] to rank possible sets of qubits on IBM_AACHEN rated by the overall error determined by the fidelities of all single-qubit and two-qubit gates involved in the circuit. The best of these qubit sets are then evaluated for their T_1 (damping) and T_2 (dephasing) times. We found that good simulation results are ensured if the T_1 and T_2 times of all qubits are above a certain threshold of around $50-100$ times the execution time of one Trotter time step. If this requirement cannot be fulfilled

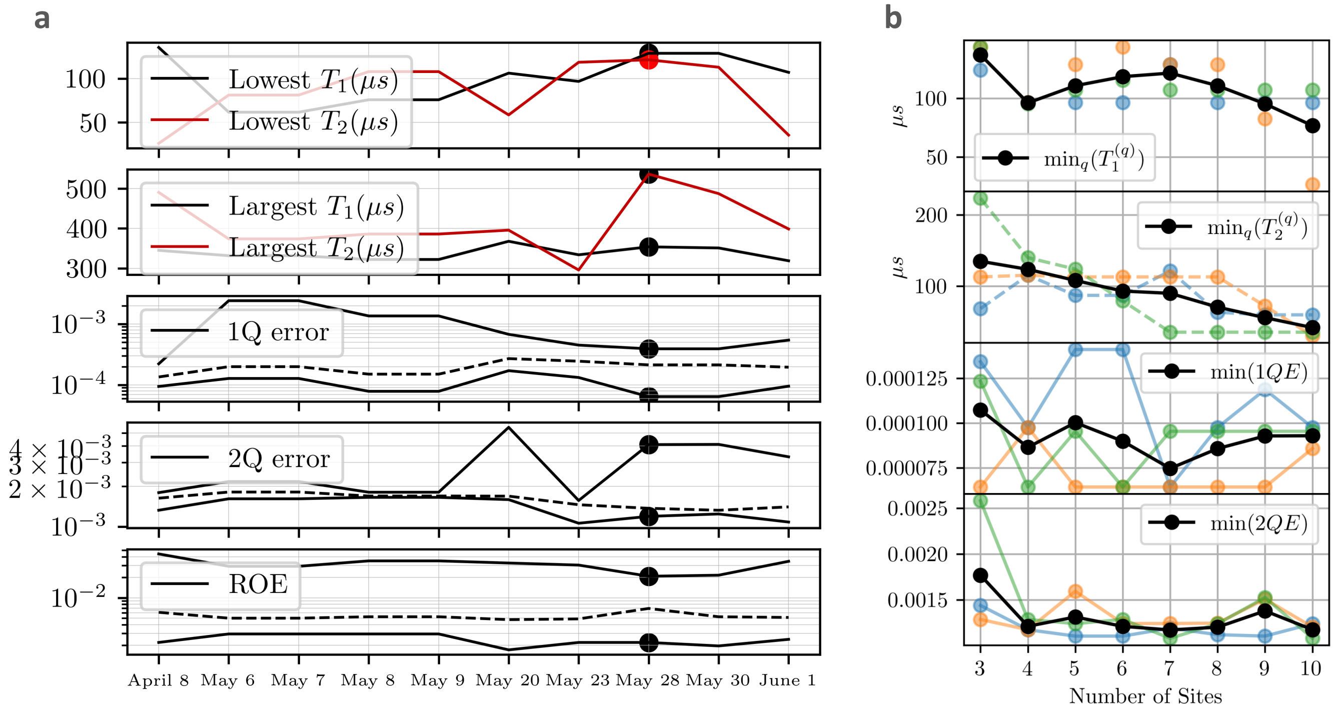

Supplementary Figure 6. **Variability of calibration data between different days. a.** For the simulations with $N = 7$ sites (14 qubits), we show the minimal and maximal T_1, T_2 times of all involved qubits, and the minimum, median and maximum for the single- and two-qubit fidelities and for the readout error. The calibration of the simulation with the overall lowest deviation $\epsilon(T)$ (see Fig. 5 in the main text) is marked with a point. **b.** Qubit properties over the number of sites N for those three runs (May 28 to June 1) which utilize the variable-angle, single-qubit R_x gates. Colored markers correspond to the three runs, while averaged quantities are shown in black.

for all qubits while maintaining good gate fidelities, then a set is picked where all system-qubits have a sufficiently large T_1, T_2 times, and then a set where at least all qubits with SWAP-gates are sufficient.

In Supplementary Table 2 we show averaged calibration data of the noise model of the qubits used on IBM_AACHEN for the smallest and largest model ($N = 3, 10$), showing that indeed the availability of qubits with sufficiently large T_1, T_2 times is less for larger systems. For the simulation presented here, the median properties for both $N = 3$ and $N = 10$ are similar, however the qubit set for $N = 10$ has some noisy outliers, affecting the overall simulation accuracy. Readout errors of the average qubit were sufficiently low to have a weak impact on the results. Our focus is the simulation of long-time evolution with as many time steps as possible (i.e circuit depth), rather than the measurement of populations very accurately at all times.

In Supplementary Figure 6 we show the variation of calibration data between different days and between different system sizes N. Panel a displays the statistics of each set of 14 qubits used for simulations with $N = 7$, demonstrating significant differences between different days. Moreover, Panel b illustrates the behaviour minimum value of each property within a set of qubits over N. The observation of Fig. 5 in the main text, that simulations with $N = 7$ show the lowest average deviation to numerical benchmarks is validated here by the overall lowest single-qubit and two-qubit errors for $N = 7$, and relatively high T_1 times. Similarly, the simulation with the lowest deviation for $N = 7$ was May 28, corresponding to the simulation with the largest minumum T_1, T_2 in the circuit and lowest single-qubit and two-qubit errors, validating our qubit selection scheme.

[1] J. Leppäkangas, N. Vogt, K. R. Fratus, K. Bark, J. A. Vaitkus, P. Stadler, J.-M. Reiner, S. Zanker, and M. Marthaler, Quantum algorithm for solving open-system dynamics on quantum computers using noise, Phys. Rev. A **108**, 062424 (2023).

[2] K. R. Fratus, K. Bark, N. Vogt, J. Leppäkangas, S. Zanker, M. Marthaler, and J.-M. Reiner, Describing Trotterized Time Evolutions on Noisy Quantum Computers via Static Effective Lindbladians (2022), arXiv:2210.11371 [quant-ph].

[3] P. D. Nation and M. Treinish, Suppressing quantum circuit errors due to system variability, PRX Quantum **4**, 010327 (2023).
